# Objective evaluation for treat to target in Crohn’s disease

**DOI:** 10.1007/s00535-020-01678-8

**Published:** 2020-03-04

**Authors:** Kento Takenaka, Yoshio Kitazume, Toshimitsu Fujii, Kiichiro Tsuchiya, Mamoru Watanabe, Kazuo Ohtsuka

**Affiliations:** 1 Department of Gastroenterology and Hepatology, Tokyo Medical and Dental University, 1-5-45 Yushima, Bunkyo-ku, Tokyo, 113-8519 Japan; 2 Department of Collaborative Medicine for Gastroenterology and Hepatology, Tokyo Medical and Dental University, Tokyo, Japan; 3 Department of Radiology, Tokyo Medical and Dental University, Tokyo, Japan; 4 TMDU Advanced Research Institute, Tokyo Medical and Dental University, Tokyo, Japan; 5 Endoscopic Unit, Tokyo Medical and Dental University Hospital, Tokyo, Japan

**Keywords:** Inflammatory bowel disease, Mucosal healing, Endoscopy, Enterography, Ultrasonography

## Abstract

**Background:**

Crohn’s disease (CD) is a chronic and destructive bowel disease; continued disease activity can lead to penetrating complications. With the recent advent of effective medications, the importance of using a treat-to-target approach to guide therapy is becoming important.

**Methods:**

In this review, we reviewed the previous evidence for evaluating CD lesions.

**Results:**

We describe ileocolonoscopy’s role in assessing disease activity, as well as recent progress in modalities, such as balloon-assisted endoscopy, capsule endoscopy, magnetic resonance enterography, computed tomography enterography, and ultrasonography. Advances in modalities have changed CD assessment, with small-bowel involvement becoming more important.

**Conclusions:**

Proper optimization is necessary in clinical practice.

## Introduction

Crohn’s disease (CD) is a chronic and destructive bowel disease, which, if left untreated, leads to penetrating complications [1–4]. Traditionally, treatment goals centered solely on symptom control, before it was recognized that many patients with CD have continued disease activity without clinical manifestations. Treatment targets have, therefore, shifted from simply relieving clinical symptoms [5] to developing objective target endpoints [6]. Appropriate therapy for a disease is based on its precise assessment. With the recent advent of effective medications, the importance of using a treat-to-target approach to guide therapy is becoming evident [8]. Mucosal healing is considered an important target of inflammatory bowel disease (IBD) therapy [9]. Since 75% of patients with CD have small bowel (SB) lesions, SB evaluation is also important [10], as the assessment of transmural inflammation and extra intestinal complications.

**Fig. 1:**
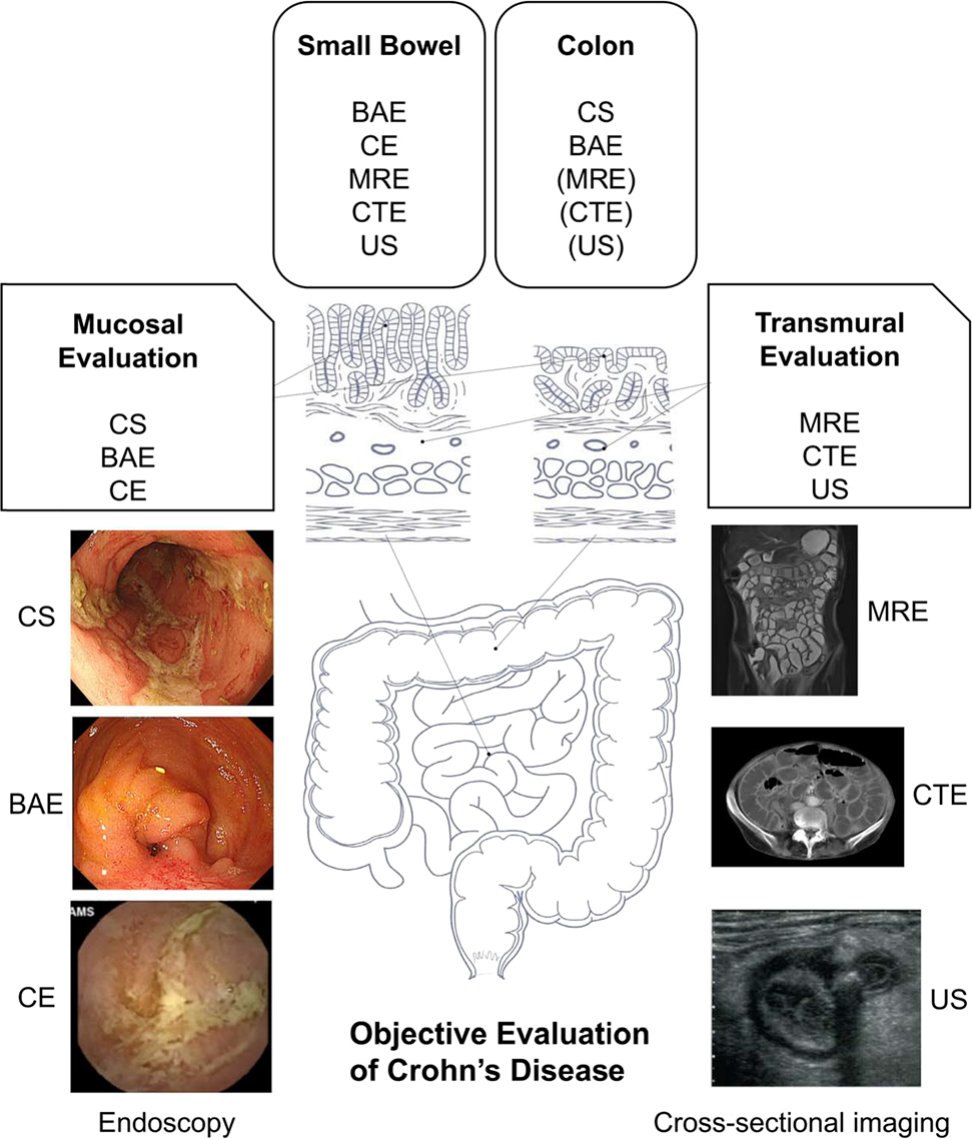
Examinations for evaluation of Crohn’s disease

In this review, we aim to describe ileocolonoscopy’s role in assessing disease activity, as well as recent progress in modalities, such as balloon-assisted endoscopy (BAE), capsule endoscopy (CE), magnetic resonance enterography (MRE), computed tomography enterography (CTE), and ultrasonography (US), which enable direct assessment of lesions deep within the SB (Fig. 1). SB follow-through is still used for evaluating SB lesion in the real world. It is, however, less sensitive and inferior to other SB imaging modalities described above [11]; and we do not state SBFT in this review. In addition, this review will not focus on diagnosis and neoplastic surveillance because those are discussed in detail elsewhere in this issue. Endoscopy and cross-sectional imaging in CD play major roles in predicting disease severity and achieving tailored patient management. Recent practices and future advances in evaluation for patients with CD are reviewed.

### Ileocolonoscopy

Mucosal healing is now considered a treatment goal in both clinical trials and clinical practice [5]. In numerous clinical trials, mucosal healing has been associated with improved outcomes in CD, including sustained clinical remission, steroid-free remission, reduced rates of surgery, and fewer hospitalizations [9, 12–17]. Patients with CD with mucosal healing have a reduced risk of penetrating complications and less need for surgery [18]. A study by Bouguen et al. found that a treat-to-target approach in clinical practice, involving endoscopic assessment of disease activity combined with adjustments to medical therapy, increased the likelihood of a better prognosis [19].

The Crohn’s Disease Endoscopic Index of Severity scoring system and the Simple Endoscopic Score for CD (SES-CD) have been used frequently in clinical trials to standardize the definition of mucosal healing [20, 21]. The main limitation of these scores is that their operating characteristics, in terms of validation, responsiveness, and reliability to assess inflammation and predict outcome in CD, are still unclear [22]. There is no validated optimal cut-off score, and disease severity has likewise quantification not yet been standardized. It is important to note that there is no validated definition of mucosal healing to date, and currently, no scoring system is used in general clinical practice. The International Organization for the Study of IBD (IOIBD) has provided a consensus definition of mucosal healing in CD as the complete resolution of visible ulcers [23]. The Selecting Therapeutic Targets in Inflammatory Bowel Disease program recommended that the absence of ulceration is used as an endoscopic target and that disease activity should be reassessed 6 to 9 months after treatment [5].

Strictures are a common and important CD complication. Indeed, 70% to 80% of patients with CD require surgery within 20 years of diagnosis, mostly due to stricture disease [24]. Endoscopy is useful in both diagnosing and treating strictures. For diagnosis, endoscopy can help differentiate between inflammatory and fibrotic strictures and guide therapeutic management. Endoscopic balloon dilation (EBD) is a less invasive therapeutic alternative to surgery for patients with strictures. Initial response rates of 65% to 97% have been reported for EBD in combined studies of both anastomotic and primary strictures [25]. A recent meta-analysis of studies evaluating EBD has reported rates of post-dilation failure, requiring surgical intervention in 18% of anastomotic strictures compared with 29% of primary strictures [26]. The combined major adverse event rate was only 4%, and the rate of perforation was only 3%. A stricture length of < 4 cm was associated with a surgery-free response [27].

Endoscopic assessment of postoperative CD recurrence is also an indispensable part of optimized management of patients with CD. Ileocolonoscopy plays a key role in evaluating for and determining the severity of, postoperative CD recurrence. Endoscopic findings of disease recurrence occur in most patients within a year of a surgical resection and frequently occur before clinical symptoms arise [28, 29]. The Rutgeerts score, a widely used schema to grade recurrence at ileocolonic anastomoses, can predict progression to clinical symptoms [30]. An IOIBD expert consensus panel recommended defining postoperative remission as a Rutgeerts score of ≤ i1 [5]. As such, endoscopic evaluation is recommended at 6 to 12 months postoperatively to assess endoscopic recurrence, with medical therapy adjustment [31, 32]. Again, the score lacks formal validation, and it is unclear which level of ileal inflammation constitutes clinically meaningful recurrence.

### Balloon-assisted endoscopy

Ileocolonoscopy is generally used to assess endoscopic lesions, but it can only assess the terminal ileum and might underestimate true SB lesions [33]. Novel BAE techniques have further increased the detection of SB mucosal lesions [34, 35] as well as allowing intubation for tissue acquisition and even therapeutic interventions [36]. Most studies have been reported retrospectively, and routine BAE’s feasibility and diagnostic utility in clinical practice for CD have yet to be established [37, 38]. However, several studies on BAE’s utility for evaluating SB CD, and endoscopic treatment for strictures, have been reported in a prospective series from Japan [39–42].

To assess inflammatory activity, a retrograde insertion is recommended because most CD lesions are located in the ileum; meanwhile, anterograde insertion should be performed for unknown upper locations [39]. SES-CD is a validated endoscopic scoring system for CD, but it is used for ileocolonoscopy, and SB lesions are not included [21]. Modified SES-CD has now been proposed, dividing the SB into three segments: terminal ileum, proximal ileum, and jejunum [40]. We have reported that BAE detected active lesions not only in the terminal ileal segment but also in the proximal ileal segment at a higher rate [39]. In addition, BAE findings have shown a poor correlation between endoscopic lesions and the Crohn’s Disease Activity Index (CDAI)/C-reactive protein (CRP). One prospective study found SB ulcerative disease in 45% of patients with clinical and biological remission, and lesions were independent risk factors for poor prognoses (relapse, hospitalization, surgery) [41]. Due to CD-related complications, BAE insertion is sometimes difficult. Takabayashi et al. reported a novel, ultrathin, single-balloon enteroscope showed adequate insertability and safety for outpatient BAE performance [43].

BAE can precisely evaluate SB strictures and also perform EBD for symptomatic patients [42, 44]. For small-bowel strictures not within reach of traditional endoscopy, deep enteroscopy can allow for evaluation and dilation in a technique similar to that used for colonic or ileocolonic strictures. A recent nationwide prospective study showed that procedure failure occurred in only 6.3% of cases, and short-term symptomatic improvement was achieved in 69.5%. In addition, adverse events occurred in 5%, and all of these improved with conservative treatment.[42]. However, this technique can be more technically complicated, given the limited size and angulation of the small bowel, and the insertion route must be determined by imaging [45].

Safety is another key for optimizing BAE in CD. The perforation rate, both with diagnostic and therapeutic BAE in CD, was similar to that observed when used for other indications [46]. Another meta-analysis showed diagnostic BAE in CD has a similar perforation rate as diagnostic BAE for all indications [47]. However, endoscopy’s invasiveness should not be ignored, and BAE has a high rate of incomplete enteroscopy [48]. Careful patient selection is a key factor in optimizing BAE use in CD.

### Capsule endoscopy

CE noninvasively visualizes the entire SB mucosa [49], and several studies have evaluated SB CE’s role in detecting known and/or suspected CD.

In suspected CD with previously negative ileocolonoscopy and/or radiologic workup results, SB CE’s diagnostic yield ranges from 26 to 71% [50, 51]. These studies had reported a higher yield in the setting of a clinical suspicion of CD, in addition to objective laboratory findings, such as anemia and elevated inflammatory markers. Despite the potentially high yield, many abnormalities found on CE are not specific to CD; thus, criteria for the diagnosis of CD by SB CE remains an area of uncertainty [52, 53].

CE in established CD has high detection rates of small-bowel inflammation [54, 55] and is sensitive for detecting lesions at previously unrecognized locations [56]. A meta-analysis has shown that capsule endoscopy is superior to barium studies, CTE, push enteroscopy, and ileocolonoscopy for detecting recurrent small-bowel CD [57]. In another meta-analysis, however, capsule endoscopy was found to have a diagnostic yield that was similar to MRE and small-intestine contrast US [58]. A recent prospective study showed video CE predicts both short-term and long-term risk of disease exacerbation [59].

Despite the potential diagnostic yield, two notable limitations of small-bowel capsule endoscopy have reduced its use in diagnosing and managing CD: the inability to obtain tissue for histologic evaluation and the risk of capsule retention. Rates of capsule retention requiring intervention have varied between studies and have been reported in 1% to 13% of patients with known CD [60, 61]. Cross-sectional imaging could help predict such strictures [62]; however, investigation with a patency capsule is recommended before CE.

### Magnetic resonance enterography

The joint European Crohn’s and Colitis Organization (ECCO)/European Society of Gastrointestinal and Abdominal Radiology (ESGAR) guidelines mentioned that MRE is an important cross-sectional imaging technique for assessing SB CD [63]. Transmural healing has been associated with improved long-term outcomes in CD [64]. MRE’s advantage is that it can acquire bowel images at multiple time-points and cinematic images to evaluate peristalsis. MRE provides both anatomic and functional information; because of higher resolution and more rapid image acquisition, it has now become the modality of choice for SB CD imaging [65, 66].

Adequate distension of the small intestine is also important for high-quality images and diagnostic accuracy [67]. Recently, several consensus statements for the optimal MRE technique in CD have been released [68, 69]. Several signs of inflammation and intestinal damage during evaluation for CD can be assessed, such as abscess, comb sign, fat edema, fistula, lymph node enhancement, reduced motility, mucosal lesions, strictures, and wall enhancement. A meta-analysis has shown that the most important signs of inflammation are wall thickness and wall T2-hyperintensity [70]. An early study on diffusion-weighted magnetic resonance imaging reported that it was comparable to gadolinium enhancement for detecting inflammation in CD [71]. MRE can provide a quantitative assessment of small-bowel motility, showing the motility of inflamed bowel segments decreases compared with non-inflamed segments. There are also advantages to using cine MRE over static imaging to investigate intestinal damage, such as adhesions, fistulas, and strictures [72].

Several disease activity scores have been proposed [73–78], including the Magnetic Resonance Index of Activity (MaRIA) [73], London [74], and Clermont [75] systems. The MaRIA, which scores wall thickness, relative contrast enhancement, mural edema, and ulcers in various segments of the gastrointestinal tract, is the most widely used [73]. The global MaRIA score was calculated as the sum of the MaRIA in the ileum and five colorectal segments: ascending colon, transverse colon, descending colon, sigmoid, and rectum. However, SB allocation is relatively small; thus, applied MaRIA can been used by dividing the small intestine into three segments: terminal ileum, proximal ileum, and jejunum. Applied MaRIA is well correlated with BAE findings [40]. The Lémann index has been proposed for intestinal damage assessment [79]. Scoring systems are often complicated and mainly used for clinical trials; however, several simple MRE scoring systems have been proposed for use in clinical practice [77, 78].

MRE could detect SB involvement and predict prognoses in patients with negative inflammation on ileocolonoscopy [80]. A recent meta-analysis has shown that the pooled sensitivity and specificity for MRE in detecting active SB CD were 87.9% and 81.2%, respectively [81]. The area under the curve (AUC) of MRE for detecting fistulas, stenoses, and abscesses was 0.936, 0.931, and 0.996, respectively. We had compared MRE and BAE findings and found that MRE was highly accurate for inflammatory SB activity, both in cross-sectional evaluation and prognostic prediction [39, 41]. Moreover, BAE and MRE showed no significant differences in terms of the AUC for predicting clinical relapse (*p* = 0.26), hospitalization (*p* = 0.96), and surgery (*p* = 0.89). For intestinal damage, however, MRE showed less sensitivity for strictures than enteroscopy [39]. Magnetic resonance-negative strictures did increase the risks for surgery compared with the patients with no SB strictures [82].

### Computed tomography enterography

Patients with CD were frequently evaluated with CTE during acute exacerbations [83]. CTE can be used as a complementary approach to identify mural healing or inflammation not detected by other methods [84]. One prospective study reported that CTE appears to be effective for monitoring activity in patients with SB CD, including patients with strictures that cannot be traversed by conventional endoscopy [85]. Another advantage is that CTE has better spatial resolution and requires a significantly shorter acquisition time, whereas MRE is currently less accessible and significantly more costly [86]. CTE provides specific, measurable parameters in evaluating the response to therapy in CD patients as well [87].

Although the anatomical resolution with CTE is excellent, routine monitoring with CTE should be weighed against the potential risks associated with radiation exposure [88]. Reducing radiation exposure as much as possible is recommended.

### Ultrasonography

International experts recommend bowel US as a tool for evaluating CD lesions in terms of complications, postoperative recurrence, and response to medical therapy [89]. It avoids radiation exposure and is, furthermore, available at bedside and associated with low costs. US techniques include Doppler US [56], with contrast agents such as contrast-enhanced US [90] and small-intestine contrast US [91, 92], and ultrasound elasticity imaging [93].

Recent interesting studies have included transmural healing under therapy as a treatment endpoint and have associated it with long-term good outcomes [94, 95]. A past study has shown an almost perfect agreement for abscesses, as well as substantial agreement for maximum bowel wall thickness, stricture, and penetrating disease [96]. Castiglione et al. [97] were among the first to define and highlight the concept of transmural healing as a bowel wall thickness of < 3 mm, assessed by bowel US. An elegant multicenter study conducted recently found that the response to therapy was associated with statistically significant reductions in bowel wall thickening or stratification, decreased fibrofatty proliferation, and increased signals on color Doppler ultrasound [98]. US is widely available and noninvasive; however, its accuracy depends on the examiner and is low in the proximal to terminal ileum region [99]. An international multicenter study reported that most US parameters used in CD showed moderate/substantial agreement [100]. Further studies will clarify the proper use of such non-ionizing radiation techniques.

The ultrasonographic subfield of multispectral optoacoustic tomography (MSOT), which is a new technique, was tested recently for the first time in 108 patients with CD to evaluate intestinal inflammation noninvasively [101]. Performing noninvasive transabdominal MSOT on patients with active CD, as well as those in remission, demonstrated that MSOT-based assessment of total hemoglobin within the intestinal wall had an excellent correlation with the endoscopic degree of inflammation. These preliminary data suggest that MSOT-based assessment of hemoglobin levels in the intestinal wall might help assess mucosal healing in patients with CD.

### Noninvasive biomarkers to assess Crohn’s disease lesions

CRP is the only blood marker used routinely in the clinic [102]. Although CRP normalization is associated with therapeutic response, CRP levels were shown to correlate only modestly with endoscopic disease activity [103, 104]. Up to 25% of patients with demonstrable endoscopic activity did not have increased CRP levels [105].

Fecal calprotectin (fC) represents an attractive biomarker, found in the stool of patients with CD since it has the advantage of increased specificity for inflammatory processes in the gut. However, it does not represent an IBD-specific fecal biomarker and is also elevated during other inflammatory or infectious processes. fC is 87% accurate for detecting endoscopically active inflammation [106]. The fC levels correlated best with colonic or ileocolonic disease, but to a markedly lesser extent with ileal disease [107]. However, a recent prospective study has reported fC showed a significant correlation with the intestinal inflammation evaluated with BAE, even in patients with the only small intestinal disease [108]. The fecal immunochemical test (FIT) is another fecal biomarker, and both FIT and fC were correlated with the mucosal status of CD [109].

A recently conducted, randomized controlled trial evaluated the therapeutic strategy of escalating therapy by tight control, based upon failure criteria defined by CRP, fC, CDAI, and prednisone use (TC group), compared with clinical management relying on only CDAI and prednisone use (CM group). The primary endpoint of mucosal healing at week 48 was met by 46% (48/122) in the TC versus 30% (37/122) in the CM group (*p* = 0.010). These results might affect future therapeutic algorithms in CD, including biomarker-based therapeutic decisions, and they underscore the ability of CRP and fC elevation to reflect active disease in CD [110].

## Discussion

There is strong evidence in favor of mucosal healing for improving clinical outcomes; however, several questions remain unanswered. In CD, mucosal healing, as defined by white-light colonoscopy, might not always reflect healing of all tissue layers, and endoscopy does not address transmural healing [7]. This leads to the question of whether more comprehensive targets should be sought. Cross-sectional imaging can acquire information about the deep layers of the bowel wall and extraluminal complications, such as abscesses and fistulas. Furthermore, colonoscopy’s ability to evaluate the extent and severity of the disease completely can be limited, particularly in the setting of more proximal SB disease. SB lesions have been observed in 70% of patients, and clinical or biochemical markers of disease activity infrequently correlate with SB inflammation [111]. Additionally, SB disease is difficult to cure with medical treatment [112], and deep SB involvement is associated with poor prognoses [113]. Evaluating SB CD will be important in the future.

Taken together, the role of objective evaluation in CD is rapidly evolving. All examination modalities have pros and cons (Table 1). Ileocolonoscopy is an important tool for evaluating mucosal healing; however, SB assessment is limited. BAE enables detailed SB mucosal evaluation, histological assessment, and endoscopic therapy, but its low accessibility and high invasiveness could be a limitation for general use. CE is less invasive, yet retention is still a critical limitation, and the clinical importance of minimal lesions is uncertain. MRE is a widely accepted examination technique and has good monitoring for SB CD, but inspectable facilities are still limited, and assessment of SB strictures shows low sensitivity. CTE has good accessibility and high spatial resolution; however, radiation exposure is a major limitation in monitoring CD. US is noninvasive and repeated assessment is suitable for clinical use, but its accuracy depends on the examiner. Noninvasive biomarkers represent valuable tools for monitoring longitudinal disease activity. We should understand them well, and optimizing how we evaluate CD lesions with SB involvement is critical to improving future outcomes.

**Table 1:** Pros and Cons of each examination

	Pros	Cons
CS	Gold standard of mucosal healing Histological evaluation Endoscopic therapy	Limited small bowel assessment
BAE	Detailed small bowel assessment Histological evaluation Endoscopic therapy	Low accessibility High invasiveness
CE	Small bowel mucosal assessment Low invasiveness	Retention of capsule
CTE	Evaluation for extraintestinal complications Good accessibility High spatial resolution	Radiation exprosure No validated score Low sensitivity for small bowel strictures
MRE	Evaluation for extraintestinal complications No radiation exprosure Widely accepted for monitoring	Limited examination facilities Low sensitivity for small bowel strictures
US	No invasiveness Possible for repeated assessment	No validated score Accuracy depends on the examiner

*CS* ileocolonoscopy, *BAE* balloon-assisted enteroscopy, *CE* capsule endoscopy, *CTE* computed tomography enteroscopy, *MRE* magnetic resonance enterography, *US* ultrasonography

## Conclusions

We reviewed objective evaluation modalities for CD. As modalities have advanced, they have changed the assessment of CD, with SB involvement becoming more important. Proper optimization is necessary in clinical practice.
